# Poetry and Neuroscience: An Interdisciplinary Conversation

**DOI:** 10.1353/con.2016.0021

**Published:** 2016

**Authors:** James Wilkes, Sophie K Scott

**Affiliations:** Durham University; Institute of Cognitive Neuroscience, University College London

## Abstract

Dialogues and collaborations between scientists and non-scientists are now widely understood as important elements of scientific research and public engagement with science. In recognition of this, the authors, a neuroscientist and a poet, use a dialogical approach to extend questions and ideas first shared during a lab-based poetry residency. They recorded a conversation and then expanded it into an essayistic form, allowing divergent disciplinary understandings and uses of experiment, noise, voice and emotion to be articulated, shared and questioned.

## Experiment

JW:
Shall we start by talking about what experiment means in each of our fields?^1^ I think about experiment as a process of trying things out, and so when I say I write “experimental poetry” what I generally mean is that the outcome isn’t determined in advance, and I’m prepared for things to go wrong.

*To our minds this is related to the idea of the essay itself, especially in its obsolete sense, equivalent to* assay*, meaning to test or trial. Iain* [Other P-331]*Biggs, among many others, has excavated the deeper roots of the word in the Latin exagium, a weighing:* ^2^ *at this etymological depth one can feel the tug of the emergence of inductive method, of a process of weighing up probabilities rather than deducing knowledge from premises (even if for Francis Bacon himself, the essay form was, in Lisa Jardine’s words, simply a way of “projecting . . . precepts in an appealing and readily acceptable form.”*^3^*)*

*What you are reading now is a particular kind of essay: one that takes its lead from Joan Retallack, who proposes poetic experiment as “conversation with an interrogative dynamic,”*^4^ *and Brian Dillon, who advises that academic essays “become essays at the moment they aspire to be other than academic, when they sideline rigor for the pleasures of seduction and surprise.”*^5^ *This is a piece that takes as its skeleton a recorded conversation we had in late 2013, which we have then larded with commentary wherever the pressure to digress or interrogate became overwhelming. We are unsure what shape the resulting creature has, but we hope its contours are at least interesting*.

I’m often looking to try something that I haven’t done before, and I’m prepared to see what comes out of a process of making where the end product isn’t determined in advance but emerges from that process.

SS:
I suppose experiment to me has a similar sense, in that science is our way of finding out about the world. And within that there are two broad ways of doing science; you can do an observational study, where you have something that you observe, and you make that observation scientific by being formal about your observation processes, and trying to be “objective” in how you do this. On the other hand you have an experiment, where you take a situation and you manipulate that situation, and see what the effects of your manipulation are, to put it crudely. With either of these, experiments or observational studies, you’re trying to find something out, and you don’t know what the outcome is going to be. You might have a good [Other P-332]idea about which way it’s going to go, but that’s not necessarily going to be right; and there might not be any effect at all.

If I was going to give you an emotional map, experiment is a positive, exciting word for me. It’s got the connotation that there’s going to be data to look at, and I can honestly say that I’ve never stopped finding data interesting. No matter what’s happened in the experiment—how many rows there have been over the construction, how badly you think parts of it have been run—when you get some data to look at you get a bit excited. Here’s something we can look at, and there’s something it will tell me once I can interrogate it in the right way.

JW:
I like the distinction you draw between the construction of the experiment and the data, because in poetry it’s different: those things are actually joined together. You don’t present results from your experiment; the results *are* the experiment in a sense. There’s not that same separation. So for example, with the work I was doing based on the anechoic chamber, I processed the recordings that I’d made in there to make new poems, but that’s different from saying, “OK, this is my data, now how can I interpret that.”

*In June 2012, Sophie took James into UCL’s anechoic chamber, a room designed to block out external sounds and to absorb any noises made inside it, making it one of the quietest places in the world. Aware of John Cage’s experiences in Harvard’s anechoic chamber*,^6^ *he came expecting to hear something, but was surprised by the rich ecology of sounds that presented themselves as he sat alone in the room with the lights out. In a recording in which he tries to describe as closely as possible the sounds he could hear, he talks about twittering birdcalls, a wet crackling sound, and a sense of dull pressure that he seems to have experienced as auditory. This recording then became the basis for a series of poems called “Describing Silence: Six Distortions,” in which he progressively distorted these words through a variety of chance and subjective procedures, each distortion being in a sense a mishearing of the last, a way of returning echo or reverb to the driest of recordings*.

And so the kind of knowledge that’s produced out of that is an experiential one; anything that people find out about the possibilities for literature or for lived experience is known in the performance, or the hearing, or the reading of the poetry. I think that’s true for *certain* kinds of poetry, at any rate. So the kind of knowledge that comes out [Other P-333]of that is different: there’s not the same emphasis on replicability. I mean, why would you want to replicate a poem?

*With complacent ignorance, James has asked a very good question. There could be several answers to this. A famous one comes from Borges, whose character Pierre Menard, a twentieth-century “symbolist from Nîmes” rewrote, word for word, a few chapters of* Don Quixote.^7^ *This is neither transcription, claims Borges’s narrator, nor copying: instead, we might think of it as a mirroring or doubling through willed coincidence. Such a doubling is impossible, though, and not only as a matter of practical effort. Where Cervantes writes a “mere rhetorical eulogy of history,” Menard proposes an idea that the narrator judges “astounding” coming from “a contemporary of William James,” by writing exactly the same words*.^8^ *The act of duplication, in its productive futility, is utopian: Menard writes, in a letter to the narrator, that “every man should be capable of all ideas, and I believe that in the future he will be.”*^9^

*The significance of framing and the importance of context in the generation of meaning have since become critical commonplaces, though proponents of conceptual poetry have recently restated the arguments for replication. Notwithstanding the bombast with which some of these claims are made, one good argument for replicating a poem is to exploit the possibilities of writing in the age of the database. As Craig Dworkin has put it, conceptual poetry “often operates as an interface—returning the answer to a particular query; assembling, rearranging and displaying information; or sorting and selecting from files of accumulated language.”*^10^ *This therefore speaks of a desire among poets to be up-to-date, which may be terribly melancholy (and nowhere more so than in the fact that this idiomatic expression is itself hopelessly old, having first entered the language in the 1890s), but is not in itself unpraiseworthy*.

Though maybe there’s a parallel in your work—perhaps replicating an experiment is necessary but not as exciting?

SS:
It is necessary, and I think the most important replication is [Other P-334]when somebody else replicates it. We’ve just done a big study trying to replicate an effect that somebody else has published quite a few papers on; it’s all come from one lab, and we *cannot* replicate it.

*Sophie ran a study to replicate someone else’s original finding; namely, that people with training in music are better at listening to speech in noisy environments than those without musical training*.^11^ *This is a potentially fascinating and important result, and she planned take the task into a brain scanner to look at the neural basis for the effect, hoping to find how musicians’ brain responses might differ. In brief, she has not gone into the scanner yet, as she could not reproduce the original results. She found other effects—for example, musicians are better than non-musicians at discriminating small differences in pitch—so she is pretty sure that she has not simply run a poor study, but the expected effect has boiled down to a more prosaic explanation (people with higher IQs do better at listening to speech in noise). This is interesting, and she has written it up in the context of the original research*,^12^ *but if she were perfectly honest, it would have been great if the effect had been replicable and she could have gone on to look at the brain results*.

*There is a great argument going on at the moment in the social sciences about a supposed “replication crisis,” with a few high-profile results in social psychology proving to be hard to replicate, or conjectured to possibly result from unconscious biases in the experimenters*.^13^ *However it would be a mistake to assume that this is unusual: the process of science is predicated on the assumption that we make scientific progress by building and developing on past work and previous interpretations. Part of this process is the refinement and improvement of scientific methods, and this is a process that often involves either overturning or reframing previous studies’ results*.

Replications of experiments, and failures to replicate, sound a bit boring, and it is boring, in some ways, but also essential, and also fascinating. It’s as much part of science as a new experiment. It’s never an end, a failure to replicate; it opens more doors, it asks more questions. [Other P-335]

I think there’s a belief around that if you can just get everything right, you can do a perfect experiment, and then you’ve got the last word on something. And that’s not it: it’s a process, and a hundred years from now, it would be very interesting to see whether the work that’s going on at the moment in neuroscience and psychology will still be discussed in the scientific framework at all. And we have no control over that; all we can do is do the best we can with what we’ve got. You never reach an end point; you’re never right—you’re just less wrong. Many other factors drive and structure scientific careers—getting funding, getting published, getting promoted. But at the heart you’re still asking questions about the world and trying to answer them—it’s about the pleasure of finding things out, as Feynman put it.

JW:
One of the things I really like is that when you start speaking to scientists, almost as soon as you start speaking to them, you realize that there’s this sort of reflexivity within science, which is something that’s perhaps occluded in popular perceptions of science as something which proceeds by a kind of ever-more accurate cleaving to a point of truth, even if it never reaches it. Anyway that’s one position within the philosophy of science, but *actually* there’s this kind of recursiveness in the sense that you’re always referring to or working with elements from the history of your field.

*James was hand waving here to the realist position, even though scientific realists might focus their arguments less on truth than on “novel predictive success”: the extent to which a theory (or parts thereof) might make successful predictions of facts beyond those used to construct it*.^14^ *Peter Vickers has assembled and discussed twenty historical examples in which rejected scientific theories produced novel predictive success, in a paper that tries to refine the realist position*.^15^ *One interesting case is that of the eighteenth-century instrument makers who successfully made achromatic telescopes by combining two different lenses made of different kinds of glass, based on a false analogy with the supposed refractive properties of the lens and the humor of the human eye*.^16^ *In fact, as Vickers notes, the human eye suffers from exactly the kinds of “chromatic aberration” as early telescopes, which is only corrected in* [Other P-336]*the brain*.^17^ *Vickers derives a point from this concerning the difficulties of deciding which parts of a theory are involved in making a prediction; but for us, the interest comes in contemplating the ambiguous relationship between a system of knowledge, later abandoned, and its dependent but surviving invention. The usefulness of the false analogy, the train of thought that rattles loose enough to jump tracks, or the leap of connection that misfires but lands somewhere hospitable*.

Which is perhaps an obvious thing to say—it would be impossible to think of any field of understanding which doesn’t do that—but that sense of circularity or circulation shows the extent to which science is a cultural activity. And it made me think of the relationship between poetry and criticism; when you’re talking about replication and the relationship between two lab groups, it made me think of two critics interpreting a work and each trying to offer a more convincing interpretation. And if you’re a poet and a critic—even if that’s a critic in an amateur sense—you’re reading people’s work in a critical way, because you’re trying to work out what they’re doing, and you’re trying to see if the effects that they produce are also ones that you understand.

SS:
A paper’s just come out saying there’s this whole literature on visual imagery, and there’s a whole other literature on visual working memory, and everything about them is different; they use different experiments, they publish in different journals, there’s no cross talk—and are they one and the same thing? And it looks like they probably are, actually: they’ve just been called different things, but the phenomena both literatures have been addressing are the same. And that’s something that can happen entirely because of the factors that have kept the literatures apart. These factors probably include, at the individual level, a kind of silo mentality about only finding out about work that seems relevant to what one is doing oneself, and not reading more widely, as much as it’s a wider issue around different research traditions and different research communities. But if you take a step back, that’s a positive development in the literature; now people are starting to address the two in the same framework. And one of the things I’m slowly coming to realize about science, and this is probably true of other fields of human endeavor, is that the rate at which there is progression is not measured in the same units as scientific careers. So any one window that you get over your life in science, you won’t see—it’s a bit like evolution, invisible over your lifespan, but there is change going on. By progress, I don’t mean [Other P-337]some idealized, linear improvement in how people think; I just mean change in how they frame and approach questions.

JW:
In some ways we do in literature think in much longer time-scales: think about translations of classical authors, which are continually being remade. And the way the classic is treated is very interesting, because poets in a sense are undoing as much as translating these works; the question is how do you make these works contemporary, and often it’s by a complete travesty of the original.

*A travesty might be no more than a change of clothing, though you would not guess this from the accreted fears of grotesquery, debasement, and sexual impropriety that have grown around the word. Two of James’s favorite classical works reupholstered for the twenty-first century start like this: “We begin in the dark and birth is the death of us / Who said that? / Hegel / Sounds more like Beckett” (Anne Carson covering Sophocles), and “the innumerable years / full of nobbers” (Tim Atkins covering Horace)*.^18^

*On translation as “undoing”: Clive Scott bases his “centrifugal” practice of translation on the idea “that the text is constantly in search of itself; that it does not comprehend itself; that it has yet to fulfil itself.”*^19^ *This frees the translator from a fear that the source text is in need of protection, or else that contains an ultimate authority; it really was like that when we found it*.

*Scott also grounds this mode of translation historically, pegging its synesthetic tendencies to the “advent of abstraction” around 1912: an artistic development that “facilitates unhindered passage between the senses.”*^20^ *The importance of abstraction is certainly familiar to a practice of science that depends on the fungibility of information, its ability to be transformed without loss or slippage across different modes of representation (from mathematical to visual, for example). But Scott also proposes translation as a way of “outwitting . . . the alphabet” and its impoverished representational structures;*^21^ *translation here is not about the conservation of essential features but a way of making a text “[fan] out into multiple versions of itself.”*^22^ [Other P-338]

But in a sense there is an abridgement of huge amounts of time, which you as a scientist would possibly not make—I mean you wouldn’t necessarily look back to Aristotelian ideas, or an early modern natural philosopher—or would you?

SS:
Occasionally you encounter people who do, but you tend not to; the narrative around neuroscience is on moving forward, and I think that can take our attention away from what has come before, at our own risk. There’s a really good book by Willem Levelt about the history of psycholinguistics, pointing out that people have been doing psychological and scientific investigations of human language for centuries—and actually, a lot of the ideas that were around at the start have not changed very much.^23^ The methodologies change, and the tools you have available change—but we forget as much as we accrete. And these remain stories: I think humans are terrors for telling stories about something and then forgetting that that’s a version. It hardens into truth. If you look at Paul Broca, who in the accepted story was one of the first people to relate human language to a bit of the brain, it turns out loads of other people were saying the same thing—in particular Max Dax, who sounds like someone from Dr. Seuss. As Broca was the more high-status person, it stuck to him, and it’s still called Broca’s Area. Another example from Levelt’s book is the idea of box-and-arrow model building: we think of people drawing box-and-arrow models as being symptomatic of psychology in the 1980s; but you go back 100 years and they were called “diagram-makers,” and they were everywhere. So I think there’s a short-sightedness that can hurt science, although that’s perhaps how it has to be.

JW:
When you’re stuck with a problem, to what extent do you go back?

SS:
Pretty often, actually. If an experiment has gone horribly wrong, and you have a result that you didn’t expect but it’s a real result, then there’s going to be something out there that will tell you something about why it’s happened. And one of the things I enjoy doing is trying to trace back the ideas—I’ve always been concerned about people who have spent their lives doing one experimental paradigm, and I’ve always found that a terrifying thought, that you might spend your life doing one thing. I’ve always tried to do—some people would say not in a good way—too many different things. But to me, I don’t think you’ll understand the sorts of things I’m interested in just by doing one sort of experiment. So I actively enjoy [Other P-339]trying to winkle out other studies that might have found something different. I wrote a paper with Carolyn last year where we went back and reviewed a whole section of studies on a particular idea about temporal processing and reframed all the results.^24^

*Sophie is referring to a very popular idea in neuroscience that language processing—which is found in the left side of the brain in most adults—is not lateralized in this way simply because it is language, but because human speech has certain structural properties that the left side of the brain happens to be really good at processing. This is such a common view that until recently it was almost a dogma; its popularity might be because it provides a good way to rebut a Chomskyian argument that language is unique and special*.

*However, this idea has been maintained as an intriguing explanation with little to no empirical support. In her review Sophie went back to some of the original papers from which this position developed, and found that a number of critical terms have changed their meaning almost completely over time. For example, the understanding of “temporal” in the literature has changed since the early ’60s, from referring to a task that occurs in time (and thus to the question of which sound came first), to the idea that temporality is a characteristic of the sounds themselves, through to the idea that “temporal” refers to a sound that is short or transient. Science is likely to be full of such examples of linguistic drift, creating problems for the accumulation of knowledge*.

## Noise

JW:
I’ve recently been thinking about noise in terms of experiment: the artefacts, the results that don’t count as data, the junk of what you’re doing. I was talking to a musician recently about junk sound, and a genre of music that uses throwaway, garbage noise, and reclaims that as the basis for music, and I thought that some of the work I’ve been doing with you was using the junk of language to make poetry. Those elements that in conversations you throw away: the tics, the repetitions, the “umms”—which produce a really textural effect; and it was that texture I was really interested in. And in promoting those, if you like, epiphenomena of language to the level of signification. So the things where you would normally say, “This is not the information; we’re having a conversation but I’m going to [Other P-340]throw away all that extra stuff, all those bits that my conversational partner didn’t mean to say, and if I had to transcribe this conversation, or relate it to someone else, what I’d relay is the message.” That’s a sort of informational model if you like; but with the poetry I was interested in taking all that stuff—and when you record it you do actually have that at your disposal—and then promoting that into the work, to say there’s something interesting going on here.

But how does it work for you? Clearly you’re interested in noise in the sense perhaps of the emotion, that’s carried in the timbre of the voice? Something that is informational, but if you were to transcribe it, would disappear entirely. And how about the noise created by experiments? The noisiness of the data from a scanner for example?

SS:
To go back to your conversational example—

*Although Sophie didn’t pick up on James’s introduction of experimental or statistical noise, it is an important consideration in her working life. Maybe she is taking “noise” literally here because so much of her work involves acoustic noise, which she has to think about continually. But, like anyone working with data collected from humans, she frequently has to deal with the high degree of statistical noise produced through intra-individual and inter-individual differences*.

To go back to your conversational example, I’ve been thinking about this exact scenario for a couple of review papers, because there’s a lot of stuff that we do to enable us to speak together. That partly includes all these funny little noises, like the “yeah yeah” I was saying whilst you were speaking, which is called “back-channelling”—you would never remember that, and in fact I was producing it in a way that would be very hard to do under voluntary control. But that’s just one bit of it: we do all sorts of things to actually make conversation possible. We coordinate our breathing: you won’t have noticed it but we’ll have started to breathe at the same rate; we’ll have started to speak at the same rate; we’ll have converged on the pitch of our voices. There are all sorts of differences we make to speak together, and we throw almost all of that away when we remember the conversation, because none of it is to do with the content, and all of it, or much of it, is to do with making the conversation possible at all. And that’s before you even get into dysfluencies: normally produced speech is phenomenally dysfluent.

JW:
So what do you think the value of that is? That stuff that gets chucked away—I think we both share an interest in it. So why is it interesting to you? [Other P-341]

SS:
I’m interested because a lot of it is to do with actually managing the conversation itself whilst you’re doing it. So if you think about the synchronous speech we did together—

*Sophie is referring here to synchronous speech, a task where people are asked to speak together. Known as choral speaking, it is common in some parts of the world as a competitive performance for schoolchildren; it is also found universally as a part of religious ceremonies, or in other situations—such as swearing allegiance or political demonstrations—where people would like to act in a co-ordinated fashion. In the same way, all modern armies still train using synchronised marching drills, although this has not formed part of warfare for over a century. Adults are surprisingly good at synchronous speech—given the same text to read aloud they can do so to a very high degree of accuracy, and align the rhythm and pitch of their voices to make this possible*.^25^

*Reading chorally in a recent performance with two other poets, James had a slight but undeniable sense of being between bodies, his attention split between the words on the script in front of him, and the two voices to his left whose enunciations guided and fitted themselves to his own. Though as Denise Riley points out, “there’s nothing new in a notion of transpersonal individuality.” She goes on to note that “nineteenth-century German philosophy is replete with it,” while “Marx’s ‘Theses on Feuerbach’ conceived of a de-centred being.”*^26^

—We had to align a behavior that we don’t normally align with somebody else; I mean we do align, but in conversation we usually do it antiphonally, so we take turns. And if you look at the characteristics of turn-taking, people never talk over each other. They don’t interrupt each other, there aren’t long gaps; the argument is that this is because you entrain your behaviour with each other. This enables you to do smooth turn-taking, which is an absolute conversational universal: anywhere in the world conversations will have this quality, even if they’re signed.

*Now Sophie is referring to conversational speech, which is governed by several universal rules, as observed by Sacks, Schegloff, and Jefferson. These include the following: speaker change occurs; one person speaks* [Other P-342]*at a time; simultaneous speech is common but brief; transitions with no gap and no overlap are common; neither turn order nor turn size, duration or content are fixed; number of talkers can vary; talk can be continuous or discontinuous*.^27^ *These rules are features of all conversations that have been investigated to date, in both spoken and signed languages. It has been argued that alignments between talkers (such as body posture and movement, voice pitch, breath, words, accent, and syntax) make this management of conversation possible*.

So it’s interesting to me because managing the conversation is basically a different set of problems you have to solve, which you’re doing at the same time as deciding what you want to talk about and how you’re going to do it. It’s unbelievably complicated, and they’re two largely separate systems.

JW:
I’m interested in poetry as it’s experienced in performance, and a performance is a very heavily coded context in which to hear another person, especially if you’ve got a couple of performers on stage, relating to each other through improvisation perhaps, which pushes poetry toward the context of music, improvised music. But the kinds of things that are happening to an audience member as they experience that are complex, and I don’t quite know how to work them back into poetry, except perhaps through an intuitive understanding of what it’s like to be experiencing this.

SS:
One of the things I find interesting about poetry and other forms of performance is how they incorporate all these elements at once. What I mean is that in an ordinary conversation, the evidence suggests that what we do when we encode information is throw away a lot of the surface stuff, and we remember the gist of what went on, which is why we don’t remember people repeating themselves. But in real time, while you’re really there, that’s experientially very present. So if I suddenly began repeating myself, you’d be picking up on that, and your immediate understanding would be colored by it. And my impression is, a lot of what poets do, particularly when they’re writing for performance, is to capture elements of that experience overtly, and play with all of those things together. It’s not as simple as saying, “They’re playing with sounds,” but those elements *are* there in the sound. [Other P-343]

## Voice

SS:
You have a comfortable performing voice, which is almost exactly the same as your normal voice. None of it sounds forced; it sounds real, if that makes sense.

JW:
I think it does. A couple of years ago I did some work with opera singers that was absolutely incredible: when you think about opera, you think about artificiality perhaps—and there’s a discourse around a sort of “genuineness” that attaches itself to poetry, and maybe that’s why my performing voice sounds the way it does—it’s part of the field in which I work, in which you wouldn’t orate in the same way.

*Although James talks down oration here, he is not above vocal embellishment himself. One of the technologies that Sophie introduced him to was Delayed Auditory Feedback, in which one’s own voice is replayed through headphones with a slight delay. This produces extreme speech dysfluencies in people who do not normally experience them, and in recent readings he has been trying to work with this phenomenon, to perform through the breathlessness, slurring, tonal changes and stammering that DAF produces. It seems to relate to the claustrophobic history of scientific enclosure that James has been researching (sparrows and titmice suffocated in air pumps, astronauts’ spacesuits failing), and to provide a way of pushing this history, so to speak, through the body of the performer*.

But the reason why opera singing is so interesting is that an opera singer is deliberately producing an incredibly artificial sound, but the power to express emotion that comes through that is absolutely incredible. One of the most amazing things was not hearing them on stage but being sat in a practice room, and they’d just get up and sing some *Lieder* or some pieces they’d been learning, at a distance of a couple of feet from you, and the emotional charge of that experience was extraordinary.

SS:
It is. Do you know about opera singers’ formants? You know you get these spectral prominences in speech, so if I go “aaar,” “ooor,” “eeer,” the pitch of my voice is the same, but I’m changing the shape of my mouth to change where the formants are, and a lot of the “identification,” to put it crudely, of speech sounds depends on the formants and how they change. What opera singers do—what we consider to be opera singing—involves introducing another formant. Some people argue that it’s two formants joined together: nonetheless it’s different. And it’s not different enough that you [Other P-344]can’t tell what they’re saying, but it seems to be a strategy for making the voice stand out. So it gives the voice a sort of spectral power, in addition to the volume with which they’re producing it. They have to sing against an orchestra without microphones so it’s a technique they’ve developed to make the voice separate from the other instruments—and coincidentally, from other sung voices.

So it’s an extraordinary instrument, and the more I work with voices, the more I realize speech is just one thing we can do with it. The beatboxers we’ve been working with can do ridiculous things! They can produce three separate sources of sound at once, and according to traditional phonetics that’s not possible, you can only do one. We only know this because we took this guy into the anechoic chamber and we got a laryngograph on him, so we were measuring the sound he was making at his larynx separately from what we were recording at his lips. And he’d start a buzzy sound at the larynx, and then seamlessly pick it up at the lips, and then produce these nasal harmonics, and he’s independently varying them all. So this gives you polyphonic music because you’ve got these three different voices going on.

There are several theories about how we evolved our voices, our speech,^28^ but it’s not clear what the evolutionary advantages of some of these changes, like the domed roof of the mouth, were before we were actually speaking. We don’t really know why we’ve got these neat little mouths, which happen to be good for speaking with. It could be that the beatboxers and the singers, and all the other things you can do with your voice, can tell us more about this. We really enjoy expertise and what people can do with their voices—maybe that’s what drove it. Kind of like a peacock’s tail.

If you look back at the history of swimming in this country, we didn’t have front crawl until relatively recently. We got front crawl from the Native Americans: there was a swimming competition in the Thames, and the Native Americans won because they did front crawl, which was considered to be very inappropriate, and terribly wrong and splashy.^29^ Now I would like to assert that we didn’t evolve to do crawl—crawl is just a phenomenally good use of the shape of our bodies and the way our arms move. Maybe that’s what speech [Other P-345]is: speech is just an extremely useful way of using what we’ve got to express human language, in the same way that our hands didn’t evolve to do sign language, but that’s an extremely good use of them for communication. Maybe something entirely different has driven the evolution of this structure, but because we can talk with it we think that’s why we’ve got it.

JW:
There’s a variation of that with poetry and voice. Voice in the physical sense is obviously the carrier through which a lot of people experience poetry; but it has these multiple harmonics of meaning in it, and people talk about voice in a way that’s metaphorical as soon as you say it. It implies all sorts of things to do with style: and that’s a productive confusion, in a way, between voice as physical and voice as style of writing. People talk about “voice” as if that’s something you need to discover, and what that really means is developing a style that’s distinctive. So it has an interesting psychological power or value ascribed to it—and once you start to talk about using quotation or found material, this “voice” then becomes a collage or composite of all these different elements.

SS:
I think voices always are. I was watching a PhD upgrade last week, and halfway through it struck me how much the student was speaking like their supervisor. Almost exactly the same, and I thought there’s so much aspiration that gets folded into your voice—who you’d like to be like—because it’s an action, it’s not something you’re just passively emitting.

## Emotion

SS:
One of the things I’m interested in is the way you express emotion in your voice. Because your voice is a thing that you’re doing, it can express your emotion in a really unavoidable, physiological way. It’s much harder to keep a poker face with your voice—there’s no such thing actually. In the grips of strong emotion your body changes, and the fight or flight reaction can affect your voice: you get that wobbly terror sound that you can’t do anything about. Every so often you get someone being interviewed on live radio who’s got that wobble going on, and you can tell they’re terrified.

And other things like laughter or crying go into direct conflict with your speaking—they’re actually using the same things. When you start getting spasms in your chest wall associated with crying and laughing, those are the same muscles you use to speak.

And then there are the vocalizations you can produce quite involuntarily when you’re in an emotional state. When we were having a rodent episode at home, I thought a mouse had run across my foot—for no reason other than I knew there was a mouse somewhere [Other P-346]in the flat—in fact a hairgrip had fallen on my foot—and I *properly* screamed. And I thought, this is ridiculous, even if it were a mouse running across my foot, I’m not even scared, but the energy and terror in the house was so high, everyone was ready to go. Historically, there’s an argument that says sound is used throughout nature as a way of signalling alarm^30^—if you think about it, very few animals emit light as a way of communicating information because you can just rely on the fact that if there’s daylight stuff will just bounce off you and you can look brilliant with your iridescence or what have you—and actually the production of noise is associated with alarm calls across anything that’s animate. Because it can get around corners—it can work in the dark; it can work under forest canopies or in a burrow—you don’t need to see each other. So my screaming when something ran across my foot is going straight back to a very ancient evolutionary principle, that that’s a good way of indicating alarm.

JW:
And that presents a problem, both for scientists and for writers. Because it’s so obvious when someone is genuinely emotional: like you say, you hear it in the voice. And if you don’t hear it in the voice—I was thinking about the experiments you’ve done with social laughter, fake laughter, which people can identify instantly. The dominant emotion in any poetry reading is low-level anxiety, in the voice of the reader, so how would you create a sense of terror? I don’t think you could; I mean maybe you could train yourself, and that’s part of an actor’s training, but for people who simply perform their work it’s a problem. It’s not accessible to writing and re-performance, unless you terrify yourself or make yourself ecstatically happy. Those kinds of emotions are very difficult, and I know you’ve had similar difficulties ethically and practically.

SS:
All of the work on the recognition and the perception of emotions, all of that’s done with posed emotions. And Paul Ekman’s work on faces wasn’t even getting people to pull emotional expressions; he was just posing their muscles.

*Paul Ekman built on the work of people like Darwin who had predicted that some emotional states would be “basic” emotions, universal and common across mammals, with distinct expressions and neural bases for each emotion. Thus anger looks and sounds like anger in many different animals. Ekman developed a system for decoding and describing facial expressions, the Facial Affect Coding System, and his famous facial emotion stimuli are based on constellated positions of the facial* [Other P-347]*musculature, rather than on people posing particular expressions of emotion*.^31^

So he wasn’t saying, “Look scared”—he was saying, “Raise your eyebrows.” And it’s a criticism I’ve always had: these emotions are all posed; why don’t you take things from films? And yet the problem I have as a scientist is that the sounds on films are not acoustically isolated. So after I started working with positive emotions,^32^ I started looking again at this, as it occurred to me that ethically we could get people to start really laughing in an authentic way, and compare this to posed laughter, and we don’t have to hurt anybody or frighten anybody or disgust anybody so much they make a noise.

And we’ve found that there really are interesting differences between involuntary, spontaneous laughter and more posed, controlled social laughter, though they are both used quite frequently across different kinds of interactions. Social laughter, for example, is not just a weak form of “real” laughter, but seems to have its own characteristics—for example, often being quite nasal. This work has allowed us to start to glimpse a lot more of the complexity of laughter in communication and interactions. And we’re now starting to ask similar questions about other emotions, such as sadness. In infancy and childhood, crying is used in a highly controlled way to manage interactions, but this is much less common in adulthood—how does this affect the ways that crying is perceived across different age ranges, for example?

JW:
I think the historical dimension of that is fascinating too. I was just reading Sianne Ngai’s book about “ugly feelings,” which is precisely about those slightly ignoble passions, so not fear or rage or weeping, but paranoia, envy.

*While Ngai does deal with disgust—one of the so-called “basic” emotions—in her afterword, she writes that although it is “the ugliest of ‘ugly feelings,’” it is “an interesting exception” in that it is not liable to produce “the confusions between subject and object” like the other feelings she discusses*.^33^ *These feelings are all to do with frustrated* [Other P-348]*agency—a mode of being in the world that is particularly characteristic of what Ngai calls, in a formulation borrowed from Adorno, the “‘administered world’ of late modernity.”*^34^

Because I think one of the things about the crying is that surely it makes a demand on you—it makes a really strong demand. So people rated it as very visceral?

SS:
We have found that if we present people with “real” versus posed crying (we had people really weeping in the anechoic chamber to get these sounds), participants rated real crying as being very arousing, very visceral, but if you asked them, “Is that real crying, or is it posed?” they’ll say posed. Whereas they’re quite happy to say that real laughter is real laughter. Our participants may be flat out refusing to believe that we actually made people cry.

I’m painfully aware that when I talk about emotion I’m working in the area of the basic emotions, and it can be crude. The basic emotions are fear, anger, surprise, sadness, disgust, and happiness—and happiness probably means laughter. And relief may well be another basic emotion, but things like pleasure, achievement, triumphant sounds—they are not. Although I think we are running into display rules around pleasure. I think people everywhere are very uncomfortable saying that any sound uttered by a man is a pleasure sound. So things like envy aren’t really addressed at all in that kind of context.

JW:
And I think those are the kind of things where you’re not talking about the voice so much, but you are talking about the things that poetry, in its sense as a way to articulate thought through a precise combinations of sounds and words, can actually get at in quite a subtle way. You can write a poem that, if you like, embodies envy, much more easily than you can write a poem that embodies terror. Switching genres here, a horror story, or a ghost story, is horrifying only to the extent that you project yourself into that imaginative scenario. You don’t get scared by hearing a ghost story, you get scared by imagining it, don’t you?

*If literature does provide a powerful mode through which to examine minor feelings, it might not only be for the prosodic reasons gestured at here through James’s talk of “precise combinations of sounds and words,” but for historical reasons too. To return briefly to Sianne Ngai, she suggests that literature is particularly apt for “theorizing social powerlessness”—the kind of powerlessness at play in feelings like anxiety*, [Other P-349]*irritation or paranoia—because, like them, it results from a “situation of restricted agency” brought about by its formation as an autonomous, and therefore politically ineffectual, sphere of activity*.^35^

*But as in our conversation, we would like to range beyond the confines of literary language, even if we cannot do more than sketch possibilities here. Denise Riley suggests that language as a whole is “radically historical, and not as any secondary or superstructural effect—but immediately so.”*^36^ *She asks if the “robust historicity” of language might not “also dwell in its emotionality, which lives in it, and which . . . is not something secondary or expressive of an inner thought sunk well below the linguistic skin.”*^37^ *This is an exciting, disturbing proposition: language is not (or is not only) the emotional speaker’s expressive vehicle, but discharges its own affective, galvanic shocks; it is a fleshly thing for Riley, something she endows with skin and “musculature” in the form of syntax*,^38^ *and in a later work imagines to have “its life as internally as any other human tissue.”*^39^ [Other P-350]

## Footnotes

1
For discussion of the significance of dialogues and collaborations between scientists and non-scientists as elements of scientific research and public engagement with science, see NowotnyHelgaScottPeterGibbonsMichaelRe-Thinking Science: Knowledge and the Public in an Age of UncertaintyCambridgePolity Press2001 PhillipsLouiseAnalysing the Dialogic Turn in the Communication of Research-Based Knowledge: An Exploration of the Tensions in Collaborative ResearchPublic Understanding of Science201201180100

2

BiggsIain Essaying Place: Landscape, Music, and MemoryProcess: Landscape and Text BraceCatherineJohns-PutraAdeline Amsterdam Rodopi 2010 149 171 reference to p. 155.

3
JardineLisaFrancis Bacon: Discovery and the Art of DiscourseCambridgeCambridge University Press1974228

4

RetallackJoan What is Experimental Poetry & Why Do We Need It?Jacket32 4 2007http://jacketmagazine.com/32/p-retallack.shtml.

5

DillonBrian Energy and Rue: An Essay on EssaysObjects in This Mirror: EssaysBerlin Sternberg Press 2014 349 359 quote on p. 349.

6

CageJohn Experimental MusicSilence: Lectures and WritingsLondon Calder and Boyers 1968 7 12 reference to p. 8.

7

BorgesJorge Luis Pierre Menard, Author of *Don Quixote*Fictions BonnerAnthony KerriganAnthony London John Calder 1985 42 51 quote on p. 47.

8
Ibid., p. 49.

9
Ibid., p. 51.

10

DworkinCraig The Fate of EchoAgainst Expression: An Anthology of Conceptual Writing DworkinCraigGoldsmithKenneth Evanston, IL Northwestern University Press 2011 xxiii liv quote on p. xlii.

11
Parbery-ClarkAlexandraSkoeErikaLamCarrieKrausNinaMusician Enhancement for Speech-in-NoiseEar and Hearing30620096536611973478810.1097/AUD.0b013e3181b412e9

12
BoebingerDanaMusicians and Non-Musicians Are Equally Adept at Perceiving Masked SpeechJournal of the Acoustical Society of America137120153783872561806710.1121/1.4904537PMC4434218

13
See the special issue of Social Psychology4532014 FrithUtaFrithChris A Question of Trust: Fixing the Replication CrisisGuardian5 28 2014http://www.theguardian.com/science/occams-corner/2014/may/28/question-trust-fixing-replication-crisis-experimenter-reputation.

14
MusgraveAlanEssays on Realism and RationalismAmsterdamRodopi199956

15
VickersPeterA Confrontation of Convergent RealismPhilosophy of Science8022013189211

16
Ibid., 198–201.

17
Ibid., 200.

18

Sophokles Antigonick CarsonAnne Tarset, Northumberland Bloodaxe Books 2012 n.p.; AtkinsTimHoraceHoraceOakland, CAO Books20071

19

ScottClive From the Intermedial to the Synaesthetic: Literary Translation as Centrifugal PracticeComparative Critical Studies8 1 2011 39 59 quote on p. 40.

20
Ibid.

21
Ibid., 51.

22
Ibid., 40.

23
LeveltWillemA History of Psycholinguistics: The Pre-Chomskyan EraOxfordOxford University Press2012

24
McGettiganCarolynScottSophieCortical Asymmetries in Speech Perception: What’s Wrong, What’s Right and What’s Left?Trends in Cognitive Sciences16520122692762252120810.1016/j.tics.2012.04.006PMC4083255

25
CumminsFredPractice and Performance in Speech Produced SynchronouslyJournal of Phonetics3122003139148

26

RileyDenise Time Lived, Without Its FlowLondon Capsule Editions 2012 78 n. xix.

27

SacksHarveySchegloffEmanuelJeffersonGail A Simplest Systematics for the Organization of Turn-Taking for ConversationLanguage50 4 1974 696 735 esp. pp. 700–701.

28
E.g., HockettCharles FThe Origin of SpeechScientific American20331960889614402211 CorballisMichael CFrom Hand to Mouth : The Origins of LanguagePrinceton, NJPrinceton University Press2002 LiebermanPhilipThe Biology and Evolution of LanguageCambridge, MAHarvard University Press1984

29

MasonPaul Heroes of Swimming: Captain Matthew WebbGuardian10 10 2013http://www.theguardian.com/lifeandstyle/the-swimming-blog/2013/oct/10/swimming-captain-matthew-webb-swim-the-english-channel.

30
CheneyDorothy LSeyfarthRobert MHow Monkeys See The World: Inside The Mind Of Another SpeciesChicagoUniversity of Chicago Press1990

31
EkmanPaulSorensonE RichardFriesenWallace VPan-Cultural Elements in Facial Displays of EmotionScience164387519698688577371910.1126/science.164.3875.86

32
SauterDisaScottSophie KMore than One Kind of Happiness: Can We Recognize Vocal Expressions of Different Positive States?Motivation and Emotion3132007192199

33
NgaiSianneUgly FeelingsCambridge, MAHarvard University Press2005335

34
Ibid., p.1.

35
Ibid., p.2.

36
RileyDeniseThe Words of Selves: Identification, Solidarity, IronyStanford, CAStanford University Press200036

37
Ibid.

38
Ibid., p. 3.

39
RileyDeniseImpersonal Passion: Language as AffectDurham, NCDuke University Press20057

